# Intracoronary artery retrograde thrombolysis for ST-segment elevation myocardial infarction with a tortuous coronary artery: A case report and review of the literature

**DOI:** 10.3389/fcvm.2022.934489

**Published:** 2022-08-04

**Authors:** Mingzhi Shen, Haihui Lu, Yichao Liao, Jian Wang, Yi Guo, Xinger Zhou, Yingqiao Nong, Zhenhong Fu, Jihang Wang, Yuting Guo, Shihao Zhao, Li Fan, Jinwen Tian

**Affiliations:** ^1^Department of Cardiology, Hainan Geriatric Disease Clinical Medical Research Center, Hainan Branch of China Geriatric Disease Clinical Research Center, Hainan Hospital of Chinese PLA General Hospital, Sanya, China; ^2^The Second School of Clinical Medicine, Southern Medical University, Guangzhou, China; ^3^Department of Cardiology, Sixth Medical Center, PLA General Hospital, Beijing, China; ^4^Department of Cardiology, Second Medical Center, PLA General Hospital, Beijing, China

**Keywords:** myocardial infarction, tortuous coronary artery, intracoronary retrograde thrombolysis, thrombosis, reperfusion preconditioning

## Abstract

**Background:**

How to deal with large thrombus burdens of culprit’s blood vessel remains a great challenge in the treatment of acute myocardial infarction.

**Case presentation:**

A 32-year-old Chinese man was diagnosed with ST-segment elevation myocardial infarction (STEMI). Coronary angiography revealed that the distal end of a tortuous left circumflex was completely occluded by a large amount of thrombus. Cutted balloon-directed intracoronary artery retrograde thrombolysis (ICART) with urokinase led to the restoration of coronary blood flow. Because there was no obvious plaque rupture or artery stenosis in the coronary artery, it was only dilated, and no stent was implanted.

**Conclusion:**

Cutted balloon-directed ICART can be performed effectively and safely in some STEMI patients with tortuous coronary vessels and large thrombus. (REST or named ICART ClinicalTrials.gov number, ChiCTR1900023849).

## Background

Intracoronary thrombosis is a great challenge and can cause no-reflow, slow-flow, malignant arrhythmia, and other adverse cardiac events in patients with ST-segment elevation myocardial infarction (STEMI) (1–3). Without a benefit and with even increased stroke risk, the recommended level of routine thrombus aspiration has been reduced or even not recommended (4–6). There is still a long way to go to solve the problem of intracoronary thrombosis, especially in patients with large thrombus burdens. In 2013, we began to use the intracoronary artery retrograde thrombolysis (ICART) technique combined with primary percutaneous coronary intervention in treatment of STEMI (7). This process produced microblood flow and microperfusion, which could be defined as reperfusion pre-adaptation and characterized by reduced reperfusion injury and improved blood flow. Here, we report a case of a young patient with STEMI with a tortuous coronary artery who was not suitable for thrombus aspiration or primary balloon dilation. For this patient, ICART was successfully performed to realize coronary reperfusion without stent implantation.

## Case presentation

**FIGURE 1:**
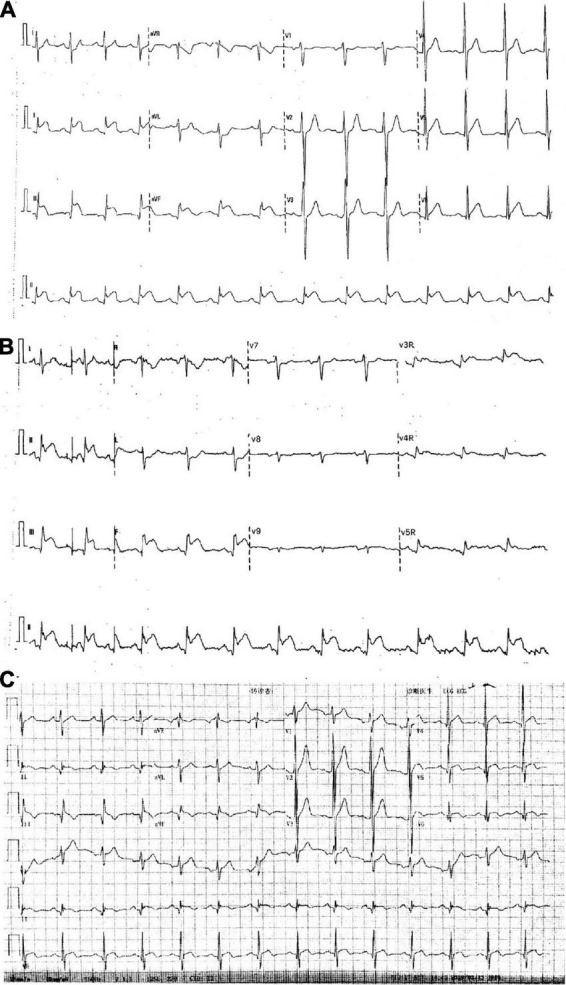
Electrocardiograms. **(A,B)** In the emergency room. **(C)** After intracoronary artery retrograde thrombolysis (ICART).

**FIGURE 2:**
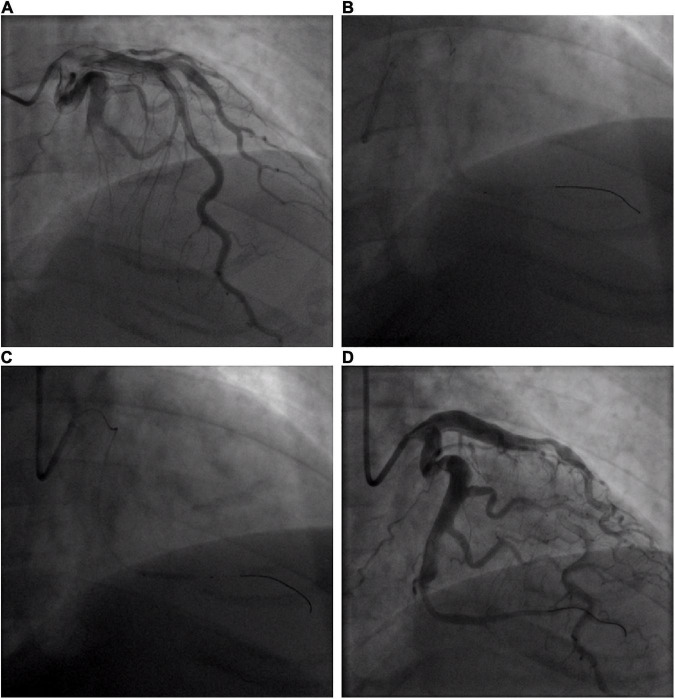
Coronary angiogram of acute circumflex artery occlusion. **(A)** Basal angiogram showing total occlusion of the left the circumflex artery (LCX) distal segment with thrombus image. The arrow shows the occlusion. **(B)** Procedure of intracoronary artery retrograde thrombolysis (ICART) through the cut balloon. The distal thrombus was gradually dissolved. The fine arrow shows the tip of the cut balloon, and the coarse arrow indicates the thrombolytic agent with contrast agent to fill the occluded lumen. **(C)** The distal end of the LCX was dilated up to 5 atm with a 2 mm × 20 mm compliant balloon. **(D)** Revascularization was achieved at the distal end of the LCX without stent implantation.

A 32-year-old young man was transferred to the emergency department with sudden chest pain lasting for 80 min. He has had high blood pressure for 4 years but on no medication, and had a blood pressure of 174/113 mmHg and a pulse rate of 78 beats per minute. Electrocardiography showed ST-segment elevations in leads II, III, aVF, V3R, V4R, and V5R (Figures 1A,B). Serum creatinine was 73 μmol/L, and serum troponin T was 7.97 ng/ml. Killip classification was class I. Aspirin 300 mg and ticagrelor 180 mg were chewed just before the coronary angiography (CAG) was performed, followed by a routine antithrombotic therapy of oral DAPT (aspirin 100 mg qd, ticagrelor 90 mg bid) lasting for 1 year. The treatment was approved by Hainan Hospital of PLA General Hospital ethics committee, and informed consent was signed. The CAG showed that the left circumflex (LCX) was completely occluded by a large amount of thrombus in the distal portion (Figure 2A). A bolus of unfractionated heparin (11,250 IU) was administered intravenously. A Runthrough guidewire was advanced through the thrombus to the distal end of the occluded LCX. The distal end of a 2.5 mm × 15 mm Sprinter Legend balloon was cut off, leaving a metal marker at the tip. Then, the balloon was inserted over the Runthrough guidewire and through the stenotic section of the occluded coronary artery (Figure 2B).

A total of 300,000 units of urokinase, 15 ml physiological saline, and 5 ml iopromide were mixed, forming a 20-ml cocktail. Following this, 1 ml of the cocktail was bolus-injected through the cut balloon, which was repeated every 30 s (Figure 2B). After injection, the mixture of contrast agents and thrombolytic agent retained in the distal end of the occluded lumen, exerted its thrombolytic effect while visualizing the occluded vessels during this process. After 7 min of ICART, the thrombus in the proximal segment disappeared, but the thrombus in the distal location still existed. An aspiration catheter was used for thrombus aspiration, but it was unable to pass through the lesion because of the tortuosity of the blood vessels. The occluded segment was dilated up to 5 atm with a 2 mm × 20 mm Sprinter Legend compliant balloon (Figure 2C) at the distal portion of the LCX. Blood flow improved to TIMI grade 2. Diltiazem 200 μg was given through the intracoronary artery. The blood flow in the coronary artery was restored to TIMI grade 3 (Figure 2D). The chest pain was completely relieved, and the ST-segment elevation was resolved (Figure 1C). Intravenous infusion of tirofiban was maintained for 36 h after PCI. Oral administration of aspirin (100 mg/day), ticagrelor (180 mg/day), rosuvastatin (10 mg/day), bisoprolol (5 mg/day), nicorandil (15 mg/day), and perindopril (2 mg/day) was continued. Low molecular-weight heparin was administered subcutaneously after stopping tirofiban. No significant bleeding complications occurred after ICART. The patient was discharged 10 days after ICART. At a follow-up time of 1 year, there was no recurrent myocardial infarction, re-hospitalization, or death happened.

## Discussion and conclusion

Management of intracoronary thrombus is a great challenge of PPCI. Large thrombus burden is associated with poor prognosis, including procedural failure, abrupt vessel closure, recurrent myocardial infarction and death (8, 9). It is widely acknowledged that the primary responsibility of physicians is to clear a thrombus quickly and open occluded vessels (10–12). Although great progress has been made in antithrombotic therapy and PCI technology, a large intracoronary artery thrombus is still a nightmare for interventional cardiologists.

In order to reduce the thrombus load of patients with STEMI, many methods such as distal protection device, thrombus aspiration, and glycoprotein IIb/IIIa antagonists are used during a PPCI procedure (13–15). However, some studies, including the EMERALD, PROMISE, and AIMI trials (16), have found that distal protection devices have no protective effect or are even harmful on myocardial perfusion and final infarct area.

Intravenous thrombolysis is simple and convenient, but the vascular opening rate is relatively low. At the same time, large doses of thrombolytic drugs increase the risk of hemorrhagic events (17). Transcatheter antegrade thrombolysis of coronary artery has been tried on patients with myocardial infarction (18). The head of a thrombus is mainly composed of white thrombi; at the same time, the thrombolytic agent cannot be retained, and transcatheter antegrade thrombolysis effect is poor. Similarly, the dose of the thrombolytic agent needs full amount, which causes a large possibility of hemorrhage.

Thrombus aspiration is one of the most frequently used thrombectomy methods to deal with a thrombus in the coronary artery (6). However, studies reveal that patients with STEMI does not benefit from routine thrombus aspiration (5). Routine thrombus aspiration showed no benefits for death from any cause or the composite of death from any cause, rehospitalization for myocardial infarction, or stent thrombosis at 1 year either (19). Moreover, thrombus aspiration even increased stroke rate (4). As such, the recommended level of routine thrombus aspiration has been reduced to grade III.

We first invented the ICART technology in 2013 to treat patients with myocardial infarction in the world (7). We amazingly found that ICART can produce reperfusion pre-adaptation characterized by microblood flow and microperfusion. It is more feasible than ischemic pre-adaptation; and when compared with post ischemic treatment, it is supposed to cause less debris, thus preventing slow blood flow and no-reflow phenomena. ICART can produce microblood flow and microperfusion, which can reduce reperfusion injury. Therefore, we summarized the following experience for myocardial infarction: urgent transport (shortening the time from symptoms to balloon opening), slow opening (by ICART rather than sudden opening of blood vessels with thrombus aspiration, so as to reduce reperfusion injury), 10 minutes of reperfusion (we found that any urgent opening is harmful, whether it is ischemic pretreatment or post ischemic treatment). We speculate that ICART will reduce malignant arrhythmia, myocardial stunning, myocardial microcirculation occlusion, intracardiac hemorrhage, and myocardial infarction area, which are related to reperfusion injury.

In this case, the blood vessel was tortuous, which is different from the cases selected in our previous article (7), the proximal part was very thick, and the distal part was completely occluded, and it had a high-load thrombus. Thrombus aspiration should be performed according to the guidelines. However, the blood vessel was very tortuous, the aspirating catheter could not pass through the tortuous lesion, which was confirmed by subsequent operation. If balloon dilatation was performed directly, there would be slow blood flow or no reflow due to high thrombus load at the distal end. The following operation also confirmed this idea. We ingeniously cut off the head of the balloon (in our previous research, we used cutted balloons, they were made by ourselves and were similar to a double lumen microcatheter), put the balloon to the end through the wire, and formed a very high concentration of thrombolytic agent locally to dissolve the thrombus, which received very amazing results. There was no thrombus in the distal vascular bed. At the same time, according to the tortuous, distal, suddenly thinning vascular structure, a stent could not be implanted, which was also the prominent feature of this case (different from our previous case). In this case, ICART successfully exposed the lesion and revealed the occluded vessel before any other intervention was taken, thus guiding the following strategies including the choice of balloons. Our present case is a supplement to our previous ICART article, which shows that the ICART method is very feasible and much safer to use even in the variant occlusive vessels.

Intracoronary thrombolysis was previously conducted on patients with an ectatic coronary artery (20). The disadvantages of this method were that the blood vessel wall was destroyed during suction, the thrombus was pushed to the far end of the coronary artery, forming slow blood flow, and no reflow. The thrombus may fall to the aorta, leading to stroke. At the same time, the reperfusion injury was very obvious. After that, antegrade thrombolysis was carried out; because the thrombus structure was destroyed, the thrombolytic agent could not be retained, and the thrombolytic efficiency was greatly reduced. At the same time, the dosage of the thrombolytic agent was very large, which significantly increased the risk of bleeding. Therefore, if ICART is performed, the thrombolytic agent will first make contact with a red thrombus and stay for a long time, so the thrombolysis efficiency will be greater. ICART is an effective, feasible, and simple approach for management of patients with STEMI. Small amounts of thrombolytics may cause very high local blood drug concentrations in an occluded section, so as to open the occluded blood vessel. The process of gradually opening occluded blood vessels produces reperfusion preadaptation, which reduces the occurrence of malignant arrhythmia. By relatively thorough removal of a thrombus, the incidence of slow-flow or no-reflow was reduced. Because the occluded segment was relatively small, no stent was implanted in this case.

In conclusion, microcatheter-directed ICART may be a safe and effective alternative reperfusion strategy in the culprit vessel for STEMI associated with massive thrombosis in small and tortuous coronary arteries.

## Data availability statement

The original contributions presented in the study are included in the article/supplementary material, further inquiries can be directed to the corresponding author/s.

## Ethics statement

Written informed consent was obtained from the patient for publication of this case report and any accompanying images.

## Author contributions

HL, YL, JW, XZ, and YN carried out patient management and data collection. YTG, ZF, and JHW drafted the manuscript and edited the figures. JT, MS, and SZ performed the angioplasty. JT and LF critically revised the manuscript for important intellectual content. All authors read and approved the final version of the manuscript.

## Abbreviations

STEMI: ST-segment elevation myocardial infarction
ICART: intracoronary artery retrograde thrombolysis
PCIs: percutaneous coronary interventions
CAG: coronary angiography
PLA: People’s Liberation Army
LCX: left circumflex
TIMI: thrombolysis in myocardial infarction.
